# Psychosocial Hazards in the Workplace as an Aspect of Horizontal Segregation in the Nursing Profession

**DOI:** 10.3389/fpsyg.2018.02042

**Published:** 2018-11-16

**Authors:** Krystyna Kowalczuk, Elzbieta Krajewska-Kułak, Marek Sobolewski

**Affiliations:** ^1^Medical University of Bialystok, Bialystok, Poland; ^2^Rzeszów University of Technology, Rzeszow, Poland

**Keywords:** gender, psychosocial aspects of work, male nurse, nurse, feminized profession, psychosocial hazards

## Abstract

The purpose of this article was to assess the influence of psychosocial hazards as a factor affecting the presence of men in the nursing profession. The article refers to the topic of particularly low representation of men among nursing staff in Poland, in comparison to what similar statistics show for other countries. The aim of the study was to assess whether the psychosocial hazards in the nursing profession constitute a significant factor in the small number of men present in this occupation. In this article psychosocial hazards are considered as all the aspects of management and work organization that may negatively affect the employee's mental and physical health. The research was conducted from September 2017 to April 2018 in the Podlaskie Voivodeship (Poland). A total of 640 respondents working as nurses in inpatient health care facilities, of which 87% were women and 13% were men, were included in the study. A standardized Work Design Questionnaire for an objective assessment of work stressfulness was used as a research tool. The research has been run by a group of experts, who explained the aim and the meaning of the particular questions to the surveyed group. Afterwards, based on the answers and observations of the responders, the experts filled in the questionnaire. The results of the study show that in almost all the evaluated aspects, the nursing profession was assessed more negatively by surveyed men than women. The most negative aspects reported by women included hazards (a score of 60), complexity (58.3), and haste (50.0), while those reported by men included haste (70.0), complexity (66.7), and hazards (65.0). As a conclusion it has been noticed, that results received from the research confirm that psychosocial hazards may have significant impact on the number of men present in the nursing profession in Poland. This study also suggests that the greater psychosocial hazards experienced by male nurses in the workplace may be an effect of the particularly low representation of men among practicing nursing staff.

## Introduction

In Poland and most countries worldwide, the nursing profession is considered to be a typically female dominated occupation. Such a view is based on the conviction that women, in line with their instincts, should perform care-related tasks (Myungkeun and Keogh, 2016; Kluczynska, 2017b). Statistical data confirm that the nursing profession is dominated by women on a global scale (Main Chamber of Nurses Midwives, 2010). This is particularly visible in Poland, where men constitute a mere 2.0% of all registered nurses, while in Canada, 4.6%; in Great Britain, approximately 9.0%; in Ireland, 10.0% and in Iran, 23.0% (Whittock and Leonard, 2003; Evans, 2004; Keogh and O' Lynn, 2007; Nursing and Midwifery Board of Ireland, 2012; Abshire et al., 2017; Kluczynska, 2017a).

These statistics cause that men are more sought after in Poland and other countries as nurses than women. Additionally, working in a female-dominated profession results in men being in a privileged position, which is demonstrated, for example, by being assigned more prestigious, administrative, and better paid tasks. This is known as the glass elevator effect, which allows men to advance only because of their gender. In the case of female nurses, a glass ceiling mechanism is activated, making it difficult for women in feminized occupations to get a promotion (Lipinska-Grobelny and Gozdzik, 2012; Williams, 2013). This is known as vertical segregation, wherein it is more difficult for women to be promoted from less-prestigious and less-paid positions, such as a unit nurse, due to the feminization of the profession. Most men working as nurses are aware that their professional development could be faster and easier due to their gender, and a promotion in the nursing profession typically means doing office work and withdrawing from patient care (Kluczynska, 2012; Lipinska-Grobelny and Gozdzik, 2012; Dudak, 2016).

Most countries around the world, including Poland, have permanent nursing staff shortages. Still, relatively few men work as nurses, despite being preferred for employment (LaRocco, 2007; Eley et al., 2012; Dudak, 2016; Kluczynska, 2017a).

One of the reasons for this is the firmly rooted stereotype that a man working as a nurse is not very manly (LaRocco, 2007; Eley et al., 2012; Dudak, 2016; Kluczynska, 2017a). The decision to perform an unmanly profession affects the perception of a man in the eyes of a large portion of society. Such a man is believed to have characteristics of laziness and submissiveness, or to be self-serving, a failure, a dependent, or of so-called subordinated masculinity. In Poland, a man who chooses the nursing profession may be perceived as a person who has failed to become a doctor and experienced defeat because he was not admitted to study medicine. Another unflattering stereotype prevalent in Poland is the perception that men working as nurses do it as secondary work, which due to the 12-h shift system enables men to do real men's work in the time between shifts (Kluczynska, 2012, 2017a; Dudak, 2016).

Undoubtedly, the prevailing stereotypes are not the only reasons for the low representation of men in the nursing profession. Another important factor influencing this issue can be the psychosocial hazards affecting this professional group, the nature, and the intensity of which may vary depending on the employee's gender (LaRocco, 2007; Eley et al., 2012; Kluczynska, 2017a).

Work under stress and psychosocial hazards corresponds to an increase in employee absenteeism loss of productivity, and high costs of health and social care (European Agency for Safety and Health at Work Topic Centre Risk Observatory, 2007). Psychosocial hazards in the workplace can be described as the aspects of work organization and management that may negatively affect the employee's mental and physical health (Cox and Cox, 1993). In the literature, there are many divisions of psychosocial hazards that consider the negative work environment determinants, also known as stressors. The classifications are usually based on theoretic models of stress or empirical data (Leka et al., 2010; Cox et al., 2016). A dynamic work environment is prone to developing existing or new hazards such as mobbing, aggression, and the introduction of new technologies (Hanna and Mona, 2014; Jamali and Ayatollahi, 2015).

No uniform classification of workplace hazards has been done so far in the scientific literature. The most common hazards classification pertains to the context and content of the work.

Hazards regarding the work context such as ambiguity and role conflict, lack of scope of control and decisions, threats, responsibility, and negative family relations resulting from a work–home conflict cause a decrease in work satisfaction (Jamali and Ayatollahi, 2015), leading to exhaustion, fear, low self-esteem, and professional burnout, finally resulting in leaving the profession (Gurková et al., 2013; Yamaguchi et al., 2016). Poor organizational culture, improper communication at all organizational levels, lowering employees' self-esteem, and mobilizing them to negative rivalry constitute sources of stress and contribute indirectly to inducing many diseases of somatic and mental bases.

In addition, work-related hazards resulting from the work context, such as quantity and quality hazards (Hamaideh et al., 2008), work complexity, work schedule, work environment and equipment lower self-control can lead to making medical errors as well as accidents or injuries leading to decreased productivity (Donnelly, 2014). Night shifts interfere with the human circadian rhythm, leading to frequent poor physical and mental health symptoms such as sleep problems and gastrointestinal disorders. Shift work generally increases morbidity and disease-related absences as well as disrupts employee functioning in family and social life (Harrington, 2001; Pilcher et al., 2003).

The aim of this study was to assess if the psychosocial hazards in the nursing profession, affecting men and women to varying degrees, may constitute a significant cause for the small number of men present in this profession.

## Materials and methods

The research was conducted from September 2017 to April 2018 in the Podlaskie Voivodeship (Poland). A total of 640 respondents working as nurses in inpatient health care facilities, constituting 87.0% of women and 13.0% of men, were included in the study. Participation in the study was voluntary and anonymous. The consent of the participants was obtained by virtue of survey completion. All procedures were approved by the Local Bioethics Committee of the Medical University of Bialystok R-I 002/296/2017 and R-I 002/175/2018.

## Study procedure

The study was conducted by a group of experts who were representatives of nurses and educators belonging to the nursing profession. These experts had a clear understanding of the purpose of the study and detailed knowledge about the specifics of the unit nurse responsibilities in inpatient health care facilities. The research was conducted using the standardized Work Design Questionnaire for an objective assessment of work stress, which was developed by Dudek et al. (1999). The experts explained the aim and meaning of the particular questions to the surveyed group. Then, based on the answers and observations of the responders, they filled in the questionnaire. Conducting the study using this method ensured objective assessment of work stressfulness. Objectivity, in this case, means that the assessment is independent of the knowledge of the stress experienced by the individual surveyed person, but is a result of the assessment made independently by 2–3 experts, who have knowledge about the specifics and work conditions of the given job position.

## Study group selection

The selection of the responders for the research group was conducted based on the register of nurses affiliated to the District Chamber of Nurses and Midwives in Białystok. The criterion adopted was employment in a hospital under an employment contract to work in the internal ward, surgical ward, admission room, or emergency department. Out of the group of people who have met the selection criteria, 10.0% of randomly selected women and 100.0% of men were invited to take part in the study. In total, 13.0% of the invited men decided not to participate. Participation in the study was voluntary and could be discontinued at any stage without providing a reason.

## Questionnaire description

A standardized Work Design Questionnaire for an objective assessment of work stress was used as a research tool (Dudek et al., 1999).

The questionnaire consisted of 34 statements describing each work characteristic. The statements were graded on a scale of 1–5, depending on the frequency, duration, or intensity of the given occurrence of the characteristic.

Based on the statements included in the questionnaire, one overall measure and ten specific measures of work arduousness were determined, including, unpleasant working conditions, complexity, hazards, conflicts, uncertainty resulting from the organization of work, arduousness, haste, responsibility, physical effort, and competition. The higher the score, the higher the work arduousness in a given aspect. However, the results for different measures are not comparable, because of a different number of component statements corresponding to each detailed measure (and the overall measure). Therefore, raw values of the individual measures were normalized in the range of 0–100 (with 0 indicating absence of work arduousness and 100 indicating maximum work arduousness), to allow for the comparison between work arduousness estimations in the different categories.

Based on the standards set out for the questionnaire, individuals with high stress levels due to work arduousness in the different areas were distinguished.

In the overall scale of work arduousness, three categories were distinguished: low, medium, and high.

## Statistical methods

Depending on the nature of the variables being compared, appropriate statistical techniques were used, including descriptive statistics of assessments of work characteristics in the men and women groups. Due to the asymmetry in the distribution of some measures, interpretation was based on the median value rather than the average. The Mann–Whitney *U* test was used to assess the significance of the differences between women and men.

## Results

This study included a total of 640 respondents (87.0% of women and 13.0% of men), who were employed as nurses. Most men were employed in emergency units, that is, 41.0% in admission room and 38.6% in emergency department. In the case of women, 37.5% worked in admission rooms, 33.8% in surgical units, and 28.7% were employed in medical treatment units and only 0.2% in emergency units.

Male nurses were better educated than their female counterparts. Higher education was reported by 79.5% of men and 57.7% of women. The structure of job positions did not indicate significant differences between women and men–a unit nurse was the predominant position held in both groups (78.3% of women and 85.5% of men). A total of 59.0% of male nurses and only 26.6% of female nurses were unmarried. Men employed as nurses were significantly younger than women and had shorter work experience (by an average of 8 years). This may have some impact on the comparison of working condition assessments presented in Table 1.

**Table 1:** Duration of work experience as nurse, depending on gender.

**Age and work experience [years]**	**Gender**									
	**Woman (*****N*** = **555)**					**Man (*****N*** = **83)**				
	**x**	**Me**	***S***	***c*_25_**	***c*_75_**	**x**	**Me**	***S***	***c*_25_**	***c*_75_**
Age	38.7	39	9.7	30	46	31.5	28	8.0	26	35
Work experience	15.1	15	10.3	6	24	7.0	5	7.2	2	10

*N, number of assessments; $\bar{\text{x}}$, arithmetic mean–variable average; Me, median–half the measures have lower values and half higher than the median; S, standard deviation (s)–a measure of “average” deviation from the mean value; c_25_ and c_75_, first and third quartiles*.

Table 2 shows the selected descriptive statistics of assessments of work characteristics in the group of women and men. Due to the asymmetry in the distribution of some measures, interpretation was based on the median value rather than the average. The Mann–Whitney *U* test was used to assess the significance of the differences between women and men. The nursing profession was assessed more negatively by men in almost all the evaluated aspects. The most negative aspects reported by women included hazards (a score of 60.0), complexity (58.3), and haste (50.0), while those reported by men included haste (75.0), complexity (66.7), and hazards (65.0). The median for the overall score was 40.4 among the surveyed female nurses and 48.5 among the male nurses.

**Table 2:** Assessment of work characteristics depending on the respondent's gender.

**Assessment of work characteristics (0–100 pkt)**	**Gender**						***P***
	**Woman**			**Man**		
	**x**	**Me**	***S***	**x**	**Me**	***s***	
Unpleasant working conditions	26.8	25.0	17.6	34.3	30.0	25.4	0.0742
Work complexity	59.8	58.3	16.1	66.1	66.7	15.8	0.0016^**^
Hazards	60.6	60.0	17.7	65.2	65.0	20.0	0.0740
Conflicts	24.0	25.0	16.8	35.1	31.3	21.5	0.0000^***^
Organizational uncertainty	41.1	37.5	25.5	52.4	50.0	25.8	0.0002^***^
Arduousness	44.7	41.7	19.1	53.9	50.0	19.4	0.0000^***^
Haste	52.6	50.0	23.9	67.2	75.0	22.0	0.0000^***^
Responsibility	30.8	25.0	29.0	34.6	37.5	32.5	0.3671
Physical effort	42.4	37.5	17.4	47.1	50.0	19.5	0.0171^*^
Competition	16.8	0	27.9	31.1	25	36.3	0.0002^***^
Overall score	42.9	40.4	12.5	51.0	48.5	15.9	0.0000^***^

*p–value of statistical significance calculated using the Mann-Whitney U test; ^x^, arithmetic mean–variable average; Me, median–half the measures have lower values and half higher than the median; S, standard deviation (s)–a measure of “average” deviation from the mean value*.

* *statistical relevance (p < 0,05)*;

** *strong statistical relevance (p < 0,01)*;

*** *very strong statistical relevance (p < 0,001)*.

Table 3 shows proportions of respondents (based on the point thresholds published by the authors of the questionnaire) whose ratings of the intensity of negative phenomena at the workplace were classified as high. As it can be seen for most of the considered aspects of work, a high level of negative opinions is more common among male nurses. This is particularly clear while assessing conflict, organizational uncertainty, arduousness, and haste. The characteristics that occur with the highest intensity include complexity, unpleasant working conditions, and haste for women, as well as complexity, haste, and unpleasant working conditions for men.

**Table 3:** Intensity of negative workplace phenomena depending on gender.

**High level of stress**	**Gender**				***P***
	**Woman**		**Man**	
	***N***	**%**	***N***	**%**
Unpleasant working conditions	384	68.9	62	74.7	0.2870
Work complexity	427	76.7	74	89.2	0.0100^**^
Hazards	297	53.3	50	60.2	0.2378
Conflicts	287	51.5	61	73.5	0.0002^***^
Organizational uncertainty	280	50.3	59	71.1	0.0004^***^
Arduousness	235	42.2	52	62.7	0.0005^***^
Haste	359	64.5	66	79.5	0.0067^**^
Responsibility	191	34.3	36	43.4	0.1066
Physical effort	273	49.0	50	60.2	0.0563

p–value of statistical significance calculated using the chi-square test for independence. ^*^statistical relevance (p < 0,05);

** *strong statistical relevance (p < 0,01)*;

*** *very strong statistical relevance (p < 0,001)*.

**Figure 1:**
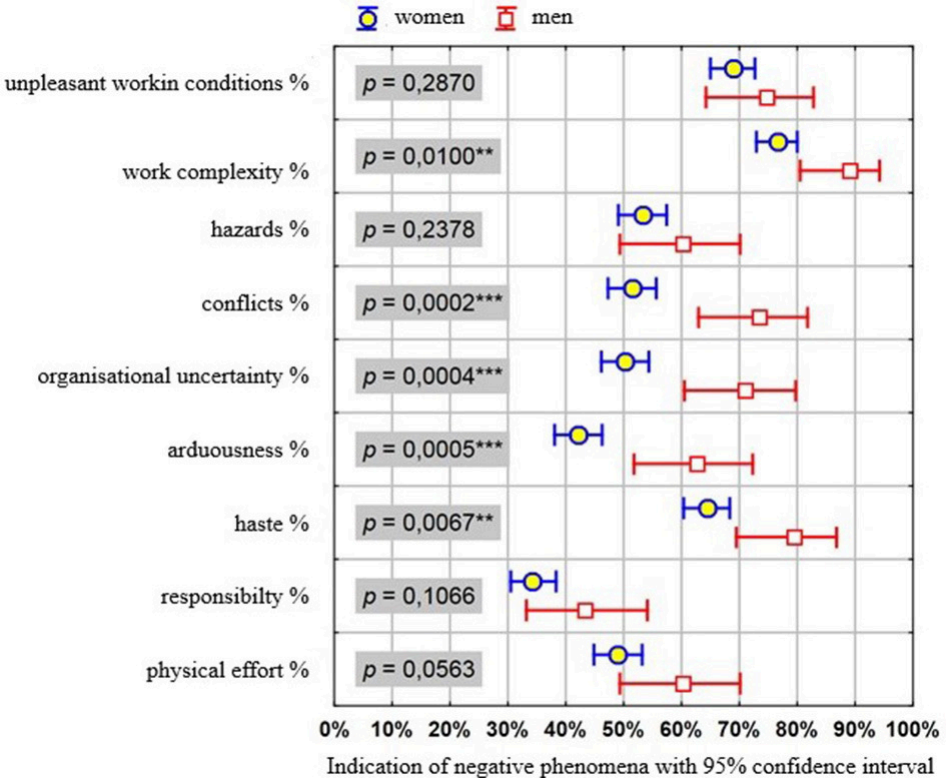
Indication of negative workplace phenomena with 95% confidence interval.

Figure 1 shows the percentage of respondents declaring a high level of negative phenomena in their professional work (along with the accuracy of estimation in the form of 95.0% confidence intervals) in the group of women and men. It further shows the result of the Chi-squared test of independence; therefore, it may be a more illustrative presentation of the results (instead of a table).

Table 4 shows a comparison between the classifications of the level of negative phenomena at work according to a summarized scale. The chi-squared test indicates statistically significant differences between female and male opinions in the intensity of negative workplace phenomena. The scale of negative workplace phenomena was described as high by the vast majority of men (85.5%) and only about two-thirds of women (67.7%).

**Table 4:** Assessment of negative workplace phenomena depending on gender.

**Overall score**	Gender (*p* = 0.0034^**^)		**Total**
	**Woman**	**Man**
Low	28 (5, 0%)	3 (3, 6%)	31
Middle	152 (27, 3%)	9 (10, 8%)	161
High	377 (67, 7%)	71 (85, 5%)	448
Total	557	83	640

## Discussion

All countries around the world, including Poland, are affected by the phenomenon of professional horizontal segregation. The percentage of men working in nursing is very low on a global scale. This is particularly visible in Poland, where men constitute a mere 2.0% of all registered nurses, while in Canada, 4.6%; Great Britain, approximately 9.0%; Ireland, 10.0%; Spain, 16.0%, and Iran, 23.0%. (Whittock and Leonard, 2003; Evans, 2004; Keogh and O' Lynn, 2007; Nursing and Midwifery Board of Ireland, 2012; Abshire et al., 2017; Kluczynska, 2017b). In most countries, stereotypes prevailing in societies are indicated as the main barrier in undertaking the nursing profession by men. What is characteristic is that the prevailing stereotypes are similar in different countries, even those that are culturally distant. Everywhere, nursing is identified with providing care, and this with being a typically women's activity. The masculinity of men who work as nurses is questioned; they are viewed as those who did not succeed in becoming doctors or those who became nurses as a result of lack of other career prospects (Neighbours, 2008; Kouta and Kaite, 2011).

Men are not only reluctant to become nurses; they also, according to research conducted by Kluczynska (2017b), much more frequently leave the profession than women in the first years of work similar inferences were obtained in the United States (Sochalski, 2002) and in South Korea (Kim and Shim, 2018). An important reason for resigning from the nursing profession is due to the workplace psychosocial hazards, which is confirmed by studies carried out by Eley et al. (2010); Cortese (2012); Rangel de Oliveira et al. (2017), and Hasselhorn et al. (2008).

In the present study, we examined to what extent the intensity of the psychosocial hazards in the workplace varies in nursing depending on the nurse's gender. On the basis of the obtained results, we found that far more men assessed their working conditions as highly unfavorable. Other studies published in the scientific literature so far have shown different results. According to a study conducted in Iran, women assessing their working conditions worse than men were in the majority; however, men constitute 23.0% of people working in nursing on the Iranian job market (Jamali and Ayatollahi, 2015). A better physical and psychophysical condition of male nurses than female nurses was found in the study conducted in Spain. The percentage of men working in the nursing profession in Spain is also significantly higher than in Poland and is over 16.0% (Rosa et al., 2013). In turn, in a study conducted in hospitals in southwestern Ethiopia, nurses' gender did not affect the degree of negative work characteristics in any way (Dagget et al., 2016).

Work complexity, as a characteristic constituting psychosocial hazards in the workplace, was assessed in our study as having the highest intensity among the tested hazards. Again, a higher incidence of this characteristic was found among men than women. Moreover, our results are also inconsistent with the results of studies carried out in other countries. Research in Andalusia (Spain) showed that men cope with work complexity a little better than women, but it should be noted that in the sample studied in Andalusia, the share of men was slightly higher than that of women, while in our study, women definitely dominated (87.0%) (Rivera-Torres et al., 2013). Similar results to the study of Rivera-Torres et al. (2013) were obtained in the research conducted in Italy (Romano et al., 2015).

The next psychosocial hazards that were indicated the most by the studied group were hazards in the workplace. In this case also, this characteristic was more intensely felt by men than women. Research carried out in Great Britain, the United States, and Iran showed that the nurse's gender did not affect the extent of perceived hazards in the workplace. In each of these countries, the number of men in the nursing professional group and the studied groups was significantly higher than in Poland (Domagała et al., 2017; Zaree et al., 2018).

Differences in the perception of conflict, a workplace hazard, to a large extent depend on individual employee's characteristics and factors such as professional experience, education, relationships with colleagues, position, and organizational climate. In our study, the potential for conflict was a characteristic with the highest discrepancy in the evaluation of intensity by men (73.5% assessed as high) and women (51.5% assessed as high). This is consistent with the results of a study in Sweden (Leineweber et al., 2014). Whereas, research from Iran showed that conflict did not depend on the respondents' gender (Najimi et al., 2012). Differences in the age and education of men and women could have affected our study results. In the studied sample, men were better educated (79.5% of men had higher education and only 57.7% of women) and younger (by 8 years), both in terms of age and work experience, than women.

Physical effort, as a psychosocial hazard in the nursing profession, was the only characteristic assessed in our study that was consistent with the results of studies from other countries such as those from Japan and Brazil (Rotenberg et al., 2008; Yada et al., 2014). This is probably due to the “He-man” stereotype, according to which a male nurse is naturally inclined to all kinds of hard physical work, because who, if not he, can lift, overturn, or bring something (Neighbours, 2008).

## Conclusions

The conducted analysis showed that in Poland, male nurses experience psychosocial hazards more severely than female nurses, and this is unlike the results of studies conducted in other countries in terms of most of the studied characteristics. Is the low representation of men in the nursing profession in Poland the reason for this? Certainly this is one of the most important reasons. It is also possible to draw another conclusion, which is a form of specific feedback, that the reason for the greater psychosocial hazards perceived by male nurses in Poland is a result of their particularly low representation in the nursing profession compared with other countries.

## Author contributions

KK: concept of the research, design of article structure, conducting of the research, review of the literature, results analysis, writing the article; EK-K: review of the literature, review of article drafts; MS: statistical analysis.

### Conflict of interest statement

The authors declare that the research was conducted in the absence of any commercial or financial relationships that could be construed as a potential conflict of interest.
