# Corrigendum: Psychosocial Hazards in the Workplace as an Aspect of Horizontal Segregation in the Nursing Profession

**DOI:** 10.3389/fpsyg.2018.02753

**Published:** 2019-01-10

**Authors:** Krystyna Kowalczuk, Elżbieta Krajewska-Kułak, Marek Sobolewski

**Affiliations:** ^1^Medical University of Bialystok, Bialystok, Poland; ^2^Rzeszów University of Technology, Rzeszow, Poland

**Keywords:** gender, psychosocial aspects of work, male nurse, nurse, feminized profession, psychosocial hazards

In the published article, there was an error in affiliation of the second author, Elzbieta Krajewska-Kułak. Instead of affiliation “2,” it should be affiliation “1.”

The authors apologize for this error and state that this does not change the scientific conclusions of the article in any way. The original article has been updated.

## Conflict of Interest Statement

The authors declare that the research was conducted in the absence of any commercial or financial relationships that could be construed as a potential conflict of interest.

